# Conceptualizing Saudi women's participation in the knowledge economy: the role of education

**DOI:** 10.1016/j.heliyon.2022.e10256

**Published:** 2022-08-17

**Authors:** Sabria Salama Jawhar, Sajjadallah Alhawsawi, Asaad Salama Jawhar, Mohmmad E. Ahmed, Kholoud Almehdar

**Affiliations:** a King Saud Bin Abdulaziz University for Health Sciences, King Abdullah International Medical Research Centre, Ministry of National Guards, Riyadh, Saudi Arabia; b Faculty of Economics and Administration, King Abdul Aziz University, Jeddah, Saudi Arabia

**Keywords:** Knowledge economy, Higher education, Female labor force participation, Human development index, Saudi Arabia, Gender gap

## Abstract

This paper is part of a project that aims to conceptualize the knowledge economy (KE) in the Saudi context, focusing on gender in relation to education, employment, human development index, innovation, and ICT. It uses a quantitative methodology. However, the used data is secondary data collected from different government and non-government sources. Different statistical analysis methods were conducted including descriptive statistics, graphs, correlation, and trend analysis. The paper found that despite the importance placed on KE and women empowerment (Saudi Vision, 2030), gender discrepancies were observed in relation to employment, innovation and ICT. Those components, according to our data, are positively correlated with the current Saudi ranking under KEI. The paper shows that although the rate of female graduates from higher education is slightly higher than male, this difference was not translated into participation in the labor market, particularly in jobs related to KE. It was also found that though Saudi Arabia's ranking under innovation and ICT was internationally low, there was a lack of public information regarding the gender dimension. This suggests that gender was not understood as a crucial factor in improving the country's ranking under those components. The paper concludes by highlighting the national innovation system's shortfalls as reflected by education, patents, and innovation. It suggests further investigations into utilizing women under jobs related to KE and calls for including the gender dimension as a variable in any future planning or studies related to the knowledge economy.

## 1 Introduction

Knowledge has been the driving force of economies and it has also become the engine of production and sustainable growth. The global interest in the transition to a knowledge economy has become a necessity to the point where knowledge has become an asset and the main component of the modern economic and social system. This transition is based on a new understanding of the role of human capital, especially female human capital, in economic development and societal progress (Olawumi, 2019).

The role of female labor market participation and its impact on economies have long been established in the literature. In this scenario, a discussion on women empowerment is analogous to a discussion on promoting economic wealth and sustainability as women have started taking the lead role in economic development (Altuzarra et al., 2019). Women's participation plays an increasingly significant role in the knowledge economy, hence it is essential to utilize this asset to the full. However, only a few studies have established a relationship between women, knowledge economy, and economic development. Most of these studies have focused on the knowledge economy and women participation as separate components (Abd Elkhalek, 2017; Nurunnabi, 2017; Arabi, 2018; Aldossari, 2020Aldossari, 2020).

In the given context, it must be noted that, like many developing countries, in Saudi Arabia, there is gender bias in the composition of most of the available educational opportunities and occupations. The ratio of women in the labor market does not match their enrollment in higher education; this gap is reflective of talent and resource wastage (United Nations Entity for Gender Equality and the Empowerment of Women (UN Women, 2018). The low rate of women participation has led to a relatively low ranking of Saudi Arabia in the World Bank's Knowledge Economy Index (KEI) (Index of Knowledge Economy, 2018). To bridge the gender gap and, consequently, accelerate the process of a transition to the knowledge economy, Saudi Arabia must gain a better understanding of the current situation and formulate a clear vision for the future. While several studies have examined the status of Saudi's knowledge economy, few have considered it from the gender perspective (Nurunnabi, 2017; Arabi, 2018; Aldossari, 2020).

This study contributes to the contemporary work by portraying a clearer picture of the relationship between gender, education, and participation in the knowledge economy. However, the researchers focused only on the five knowledge economy indicators—education, employment, human capital, innovation, and information and communication technology (ICT). It explored different data sources to investigate the prevailing knowledge economy and link it with gender participation. This study transcends the festive propaganda around female employment in Saudi Arabia. It is a data-driven study scrutinizing women participation in jobs related to the knowledge economy and connecting it to their education. It also establishes a relationship between the areas lacking a gender perspective and the country's efforts to transition to a knowledge economy.

A better understanding of the status of Saudi's knowledge economy in relation to the gender dimension will help relevant decision-makers to make informed decisions, especially regarding education's role to empower women and genderize, de-genderize, and re-genderize the knowledge economy.

### 1.1 Background

Saudi Arabia plays a very strategic role regionally and internationally owing to its economic, geographical, political, and religious position in the world. With a population exceeding 34 million, it is the biggest of the six Gulf Cooperation Council (GCC) countries. Saudi Arabia also plays a critical role in the stability of the world's economy as it possesses about one-fifth of the world's proven petroleum reserves (Peterson, 2020).

Saudi Arabia is among the first Arab countries to develop an integrated national plan to develop the knowledge economy by using information technologies (Alshumaimri et al., 2017). The Saudi government perceives this transition as a necessity dictated by the requirements of growth and the ramifications of international conditions and developments. Saudi Arabia has taken steady steps toward transforming the knowledge society and moving toward a diversified economy that is not dependent on natural resources only. The country's commitment toward this goal is reinforced by its Saudi Vision 2030 and Ninth Development Plan. Significant progress has already been made in terms of the size of investments and financial credits for several development projects such as those for general education, supporting gifted students, investing in human capital, and building qualified national capabilities in the scientific, technical, and knowledge fields (MEP, 2018).

Saudi Arabia has also attempted to include the notion of the knowledge-based economy in different phases of its national plans. For example, the Eighth Development Plan (for the period 2005–2009), focused on fundamental developments that laid the basis for transitioning to a knowledge-based economy. These developments included commencement with the implementation of the first five-year plan of the National Science, Technology and Innovation Policy and the adoption of the National ICT Plan, the National Industrial Strategy, and the Strategy and Plan for Giftedness, Creativity and Supporting Innovation. It also included the establishment of the knowledge economic city in Medina and the technology zone of the Saudi Organization for Industrial Estate and Technology Zones in Dammam, the preparation of a new strategy for higher education and for promoting privatization. The Ninth Development Plan, which focused on the 2010–2014 period, made progress toward a knowledge-based economy by focusing on education and paving the way for knowledge transfer, accumulation and generation, and its utilization in various economic and social sectors, particularly those of production and service activities. The Tenth Development Plan, covering the 2015–2019 period, focused on the diversification of the country's economy and increasing its non-oil revenue by 8.5% to reach 66% of the GDP in 2019 and, in the same year, increasing the private sector participation in the country's GDP to 50.6%. The Plan also emphasized investments in the manufacturing industries and mining sectors, assigned 372 billion riyals for infrastructural investments, and spelled out details on government spending and accountability. These initiatives resulted in investments in the knowledge economy infrastructure, such as advanced information and technology, which has become one of the mainstays of production in the country's current economy (Barkhordari et al., 2019). In this context, there has been a shift in the concept of economic competition, acknowledging that knowledge provides a competitive advantage and drives the economy.

Through these endeavors, the Saudi government seeks to enhance the comparative advantages of the economy, diversify it, and increase local productivity and competitiveness while creating commensurate employment opportunities for its citizens. Saudi Vision 2030 reflects a clear trend in pursuit of the shift toward a knowledge-based economy, incorporating efforts to diversify the country's economy through several plans, initiatives, and programs. The latest plans of Saudi Vision (2030) include the announcement of the NEOM project, which aims to build globally smart cities that depend on clean energy and provide investment opportunities in connecting continents. Vision 2030 also represents a qualitative shift in the Kingdom's policy toward dealing with economic and societal fundamentals. By setting goals for each agency in the government, the Vision also directs the components of the knowledge society toward sustainable economic goals that take the country's competitive advantages in various fields into account.

Vision 2030 encompasses the potential to promote competitiveness and sustainable social and economic growth thanks to the readiness of the science and technology systems' components, the pursuit of innovation, the completeness of the communication and information technology system, and individuals' high education and cultural levels. Utilizing and increasing the participation of female labor in the transformation process is a central focus of discourses on these development plans. Women comprise more than 50% of the country's working population, a proportion that cannot be ignored by the government (General Authority for Statistics, 2020).

### 1.2 Research aims and objectives

This study's main aims are to conceptualize Saudi women's participation in the knowledge economy and to link it with the role of education. The specific objectives of the study are as follows:

i. To identify the status of Saudi women's labor force participation in relation to the components of the knowledge economy—education, employment, human capital, innovation, and information and communication technology.
ii. To identify the relationship between education, employment, human capital, innovation, and information and communication technology and Saudi's ranking in the knowledge economy index.
iii. To explore the relationship between Saudi women's current labor force participation in relation to the knowledge economy's components and Saudi's ranking in the knowledge economy index.

## 2 Literature review

Knowledge represents one of the fundamental and most distinctive characteristics of human society. It has facilitated significant transformations covering the economic, social, and cultural aspects of life throughout history. Although the literature provides different knowledge epistemologies, it often links knowledge to various explanations and applications of the knowledge itself (Barlatier, 2004; Daňa et al., 2020). It must be noted that knowledge usually adds "massive value to economic production through increases in productivity and the application of new technologies and new ideas—both in the form of new inventions and also new applications of existing knowledge—has brought a revolutionary change to virtually all markets and sectors" (Bătăgan, 2008, 27). Thus, the rampant growth in mobile communication, Internet users, and knowledge accelerates economic development, competitiveness, and the success of the new economy (Bătăgan, 2008). The use of knowledge as a driving force for economic growth facilitates the rapid exchange of information and knowledge about services and products and reduces communication and physical distance (Bellinger, 2004). The significant role of knowledge in economic development explains the global interest in the transition to a knowledge economy, based on a new and in-depth understanding of the role of information, technology, and human capital in economic growth.

It has become common knowledge that most of the world's advanced economies are heavily dependent on the efficient production, distribution, and use of knowledge. The increased use and utilization of knowledge, information, and technology in the production process has confirmed knowledge as a factor of production and induced investment in knowledge capital. Consequently, knowledge contributes toward and develops production processes that result in job creation. The countries with the highest economic growth rates are those that have comparatively more knowledge capabilities. Other factors that justify the transition to a knowledge-based economy are the rapid growth of knowledge, increased sources of knowledge, and the emergence of new branches in science, technologies and products that enhance the dissemination of knowledge (Olopade et al., 2020).

The broad recognition of knowledge as a dominant production factor influencing economic and societal transformation and development has led to the emergence of different terms and definitions for the knowledge economy. The emergence of the numerous terms used to denote the concept of a knowledge-based economy indicates that there is no universally accepted definition (Hvidt, 2015). This study, however, draws on the following definition by the Organization for Economic Co-operation and Development (OECD):

"an economy that is capable of knowledge production, dissemination, and use: where knowledge is a key factor in growth, wealth creation, and employment, and where human capital is the driver of creativity, innovation, and generation of new ideas, with reliance on information and communication technology (ICT) as an enabler" (Organization for Economic Cooperation and Development, 1996, 9–11).

It is noteworthy that the Saudi government embraced the OECD definition of the knowledge economy (KE) in its latest development plans. This definition reflects the multiple facets of knowledge and assigns central importance to economic growth. It also shows the relationship between knowledge, job creation, innovation, and ICT use. Owing to their prevalent economic impact, the paper covers these main domains, and it does so from a gender perspective.

### 2.1 Women participation in the economy

Both genders must participate equally in improving the nation's economic capability using knowledge. In other words, working-age men and women are expected to participate equally in the KE; both genders can contribute equally, given that the advancement of the KE depends on intellectual capabilities rather than physical inputs or natural resources (Powell and Snellman, 2004). Nevertheless, the truth about many parts of the world is that men and women do not equally participate in the KE. For example, in 2018, the global rate of female participation in the labor market has been substantially lower than men's; 26.5% versus 48.5% (International Labour Organization, 2018).

The importance of women's participation in the economy has been long debated in academic research and continues to be contested to this day, mostly regarding women's effective participation in knowledge-based jobs. Several scholarly works, such as those by Fauth and Brinkley (2007), Jenkins (2005), and the World Bank (2018), argue that investing in gender equality in the workforce positively impacts economic growth and sustainability. This argument suggests that increased inclusion of women in the workforce contributes significantly to economic acceleration. Woetzel (2015) supports this argument when showing that an increase in women's economic parity can add between $12 and $28 trillion to the global annual GDP by 2025. Despite the enhanced economic growth that women's participation in the KE promises to offer, women's participation in the work force remained disparate. According to the UN Women report (2018), the global ratio of women's labor force participation is significantly less than that of males; 63% and 94% respectively. The case in Saudi Arabia is not an exception as the percentage of women's labor force participation (19%) is less than men's (64.6%) (General Authority for Statistics, 2018).

The Saudi government resolved to address this issue and expressed its commitment to increase women's inclusion in economic life across all the sectors. However, the fulfillment of this vision would require the Saudi government to amend workplace legislation and develop new ways of defining women's values as contributors to economic development. The government should also address the social norms governing women's employment and willingness to participate and focus on the nature of employment and its relevance to the labor market's demands. Concerning gender parity, Abd Elkhalek (2017) believes that the KE may be the answer to establishing gender parity in the Arab world as it contributes to expanding women's participation in the labor market by addressing the prevailing constraints. She stated that the KE "generates new job opportunities for women and changes the direction of women's participation in labor market towards more knowledge-based activities" (Abd Elkhalek, 2017, 3).

Efobi et al. (2018) examined the impact of ICT advancement on women's participation in the formal economy and found that an improvement in communication technology increases female economic participation. This indicates that the introduction of ICTs and making ICT devices cheaper and accessible to the business community and the masses can contribute toward a nation's growth and development. The telecommunication networks can also significantly influence a country's GDP though job creation and enhanced communication linkages and connectivity, among other positive effects.

### 2.2 Knowledge economy and education

Since the KE deploys knowledge as an essential tool for accumulating wealth, assuring efficiencies, boosting economic growth, and creating jobs, it is imperative to explore the relationship between education and the process of knowledge generation. There is a strong relationship between the KE and education. Educational institutions facilitate knowledge exploration, discussion, contestation, and the creation and development of knowledge applications. It is safe to assume that education produces different forms of knowledge that can be converted into capital or commodities that can advance economic growth, as argued by Barkhordari et al. (2018). Thus, the World Bank (2007, 2012) considers education and training as one of the four essential pillars of a KE. Higher education plays a significant role in providing the skills, experience and knowledge required for developing an advanced knowledge-driven economy and promoting social development. The role of higher education is not limited to teaching abstract knowledge and random skills, but it is mostly linked to the market in the most advanced economies (Sukharev, 2021). Higher education creates the demand for advanced science and technology and offers solutions to the market through innovations (Godin, 2003). A strategic collaboration between higher education and the business/market facilitates the exchange of knowledge and innovations used to increase economic competitiveness and achieve sustainable development at all levels, thereby maintaining a competitive edge in trade and industry (Salem, 2014). Policymakers view research universities as "knowledge factories for the new economy with largely untapped reservoirs of potentially commercializable knowledge" waiting to be exploited by businesses and markets (Wolfe and Bramwell, 2008, in Salem, 2014,1049). Universities represent a starting point for the growth of the KE, planning and investment in society through research commercialization, technology transfer, educational activities, and community partnerships (Bejinaru, 2017). Higher education encompasses powerful institutions capable of generating commercialized knowledge and creating valuable innovation systems. Yet, as suggested by Salem (2014, 1049), the business/market may often lack the "absorptive capacity required to fully benefit and make use of university research". The two parties must therefore collaborate to realize the objective of infusing the economy with commodities driven by cutting-edge research and innovation (Huggins and Johnston, 2009). Hence, one must be careful not to assume a linear flow of relationships between higher education and the market.

The relationship between the two parties is overly complex and fluid and involves various enabling actors (Wolfe and Bramwell, 2008). Higher education provides professional support and specialized expertise for the business/market through research and development activities (Tomlinson, 2018; Wolfe and Bramwell, 2008). The contribution of higher education to the market should not be limited to research, innovation, technology transfer and commercialization, however; it should include the workforce that higher education produces in the form of human capital (Godin, 2003). Graduates of higher education are considered an alternative form of knowledge transfer because they utilize their acquired knowledge and skills to secure employment or start their own businesses (Salem, 2014). Higher education institutions are expected to abandon their insulated "ivory towers" and engage as active actors by creating local linkages and networks to attract and retain human talent (Abel and Deitz, 2011) and, consequently, contribute to the KE. When education fails to transfer relevant knowledge into the KE, it fails to fulfill its role; this raises the question on the relationship between the outcome of knowledge transfer and employment (Hanushek, 2008). In their study done for the OECD, Machin and McNally (2007) found that the probability of college graduates participating in the labor market has increased to 73%, compared to 3% among primary school graduates.

## 3 Methodology

### 3.1 Data sources

The researchers used secondary data from various sources, including Saudi Arabia's General Authority for Statistics, the Ministry of Education, Labor, the Ministry of Economy and Planning, the World Bank, and Saudi Vision 2030. The used data were public and open-access except those obtained from the global economy website, which are paid-for. The researchers utilized time series data spanning from 2010 to 2018 for the knowledge economy indicators.

#### 3.1.1 Research variables

The variables used in the statistical analysis represent the indicators of KE's pillars. Most of the indicators were analyzed using variables broken down by gender whenever possible.

### 3.2 Data analysis

The data were entered and manipulated using Microsoft Excel software and the Statistical Package for Social Sciences (SPSS) software. Excel and SPSS software were used to run the statistical analysis of data obtained from different secondary sources. The data were organized in quantitative variables and defined in SPSS prior to the analysis process.

Since SPSS is software that can be used for both primary and secondary data, the analyses were conducted using different methods and statistical tools. That included a descriptive analysis method for mean values and graphs (line and bar charts) to present the trend of variables during the study period. Additionally, correlation analysis was used to examine the relationship between the selected variables.

## 4 Results and discussion

This section discusses the results in the Saudi context, based on the World Bank's knowledge economy framework. The first part addresses education and employment as essential indicators of the KE. The subsequent section discusses the other KE indicators—human development index (HDI), innovation, and ICT.

### 4.1 Education and unemployment

Education is one of the leading indicators of the KE, given that it promotes women's labor market participation. There is a strong correlation between education and employment (Glass, 1992); however, the impact of education on employment depends on different factors, such as the country itself (Temple, 1999). In this regard, Saudi Arabia has made significant strides in improving its education levels and reducing illiteracy rates among adults and children. Since higher education is one of the most critical preparation stages for the transition, Saudi Arabia has endeavored to increase the number of students enrolled in different schools and universities.

The rate of enrollment in tertiary education in Saudi Arabia is higher than in many other Arab countries (Barry, 2021). Although the unemployment rate was relatively low at 5.62% until 2019, this figure does not reflect the government's goals for women's participation in the economy. Qatar and Oman are two further examples of countries that emphasize gender equality and female empowerment in their 2030 visions, with education as the key means of achieving this objective (Golkowska 2017). In Qatar, 54.9 percent of women are enrolled in university education (HE), with 57.9 percent working. Given this, just 15.1 percent of women in management and senior positions are female (Global Gender Gap Report, 2021). The degree to which female education and labor market participation are connected within the Arab Gulf as a whole is revealed by looking at these numbers.

As of 2020, the percentage of unemployed Saudi women was 30.2%, and their labor market participation stood at 24.2% (General Authority for Statistics, 2020). Nevertheless, the Saudi government aims to decrease unemployment among women by 50% by 2030. This implies that the percentage of women participation in the labor market will have to increase to 30%, as expressed in Saudi Vision (2030).

**Figure 1:**
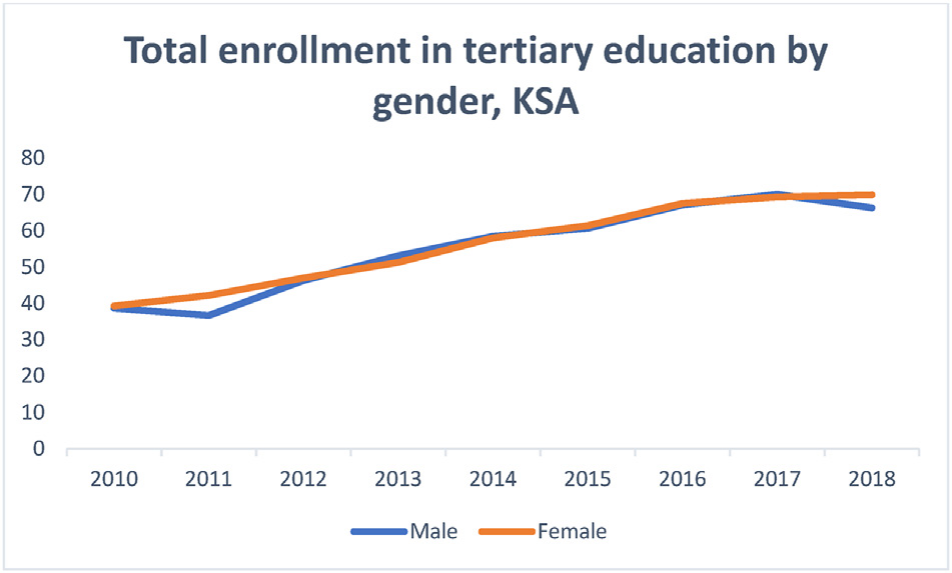
Total enrollment in tertiary education.

Source: Ministry of Education statistics (2010–2018)

Figure 1 displays the rate of enrollment in higher education according to gender during the past eight years. It illustrates an increase in Saudi women's enrollment figure, which surpassed males in 2017 and 2018. However, the difference is not significant.

**Figure 2:**
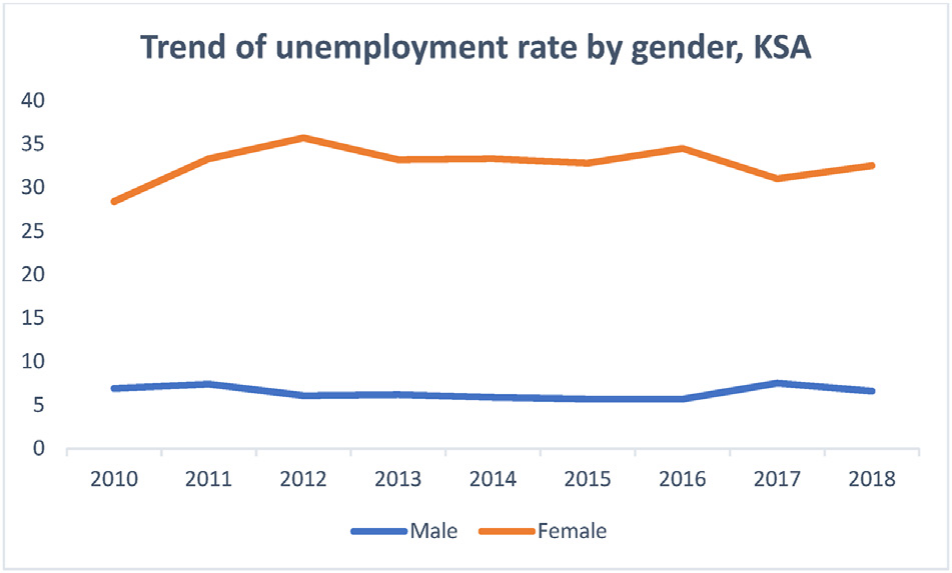
Trend of unemployment rate by gender.

Source: Labor Force Participation reports of General Authority for Statistics (2010–2018)

Despite the increase, the rate of unemployment among women graduating from higher education institutions seems to be insignificant during the same period, as shown in Figure 2.

**Figure 3:**
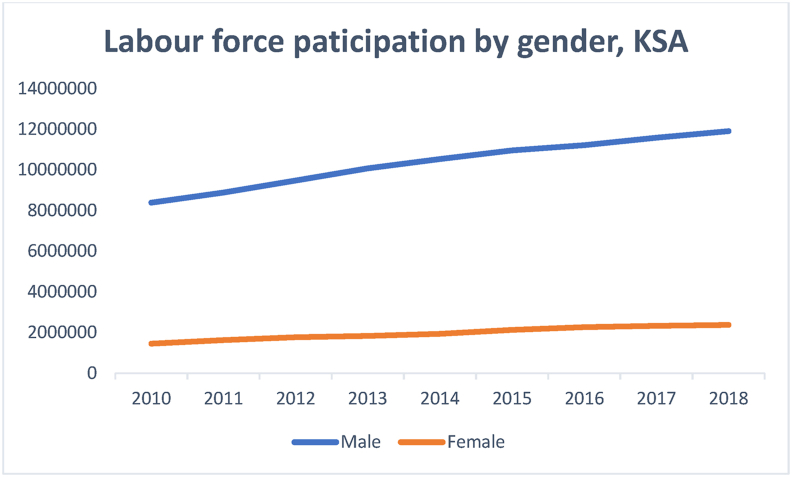
Labor force participation based on gender.

Source: Labor Force Participation reports of General Authority for Statistics (2010–2018)

Figure 2 reflects a significant gender gap between the unemployment figures for men and women. It confirms women's inability to secure employment despite having attained similar, if not higher, education levels as men. Figure 3, which shows labor force participation by gender, indicates that women enjoy much less employment opportunities than men.

Data illustrating the trend of male and female labor force participation during the sampled period confirm the statement made about the inability of females with bachelor's degrees to secure employment (Figures 2 and 3). Figure 3 reflects a significant gender difference; male labor force participation is not only substantially higher than females', but the line indicating the former reflects a continued upward trend, thereby increasing the existing gap. The pattern of female unemployment in the Saudi context is unique. It defies some of the long-standing beliefs regarding the relationship between education attained and employment secured (Becker, 1964; Glass, 1992; Temple, 1999; Machin and McNally, 2007). Although the increased access to higher education is a relevant intervention, there is an urgent need to match the education thus gained with the jobs created for women to ensure their relevance to the labor market and maximize the returns from higher education. Though discussing the Italian economy, OECD (2017) listed "increasing access to tertiary education while improving quality and relevance of skills" among the solutions for gender disparities in the labor market (OECD, 2017OECD, 2017, p.23). Bao et al. (2019), attributed gender disparity in the Arab Gulf states to factors other than education. They listed local context, power structures and social arrangements among the factors that impact women participation in the labour market (for further information see Alhawsawi and Jawhar, Forthcoming).

**Figure 4:**
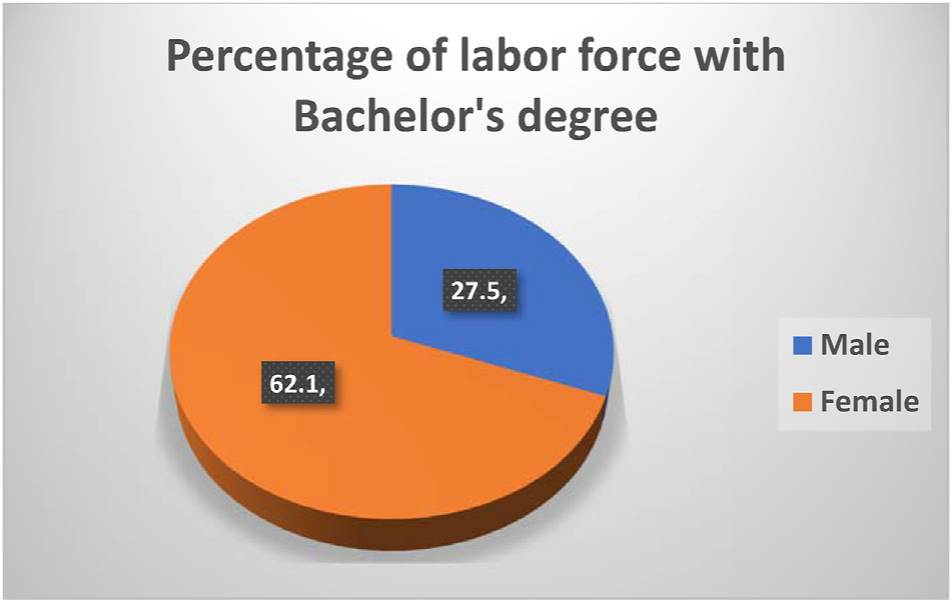
Percentage of labor force participation based on gender with bachelor's degrees.

Source: Labor Force Participation reports of General Authority for Statistics (2010–2018)

Figure 4 shows the percentage of female bachelor's degree holders involved in the Saudi labor market, compared to their male counterparts. Concerning the gender disparities, the figure shows that only 62% percent of the female bachelor's degree holders are absorbed in the Saudi labor market, which confirms Machin and McNally (2007) argument regarding the relationship between tertiary education and the labour market. However, only 27.5% of male bachelor's degree holders are enrolled in the labor market. A comparison between Figures 1, 2, 3, and 4 suggests that the Saudi labor market favors males, regardless of their educational background; this is evident from the fact that although the men dominate the market, only 27.5% of them hold bachelor's degrees.

**Figure 5:**
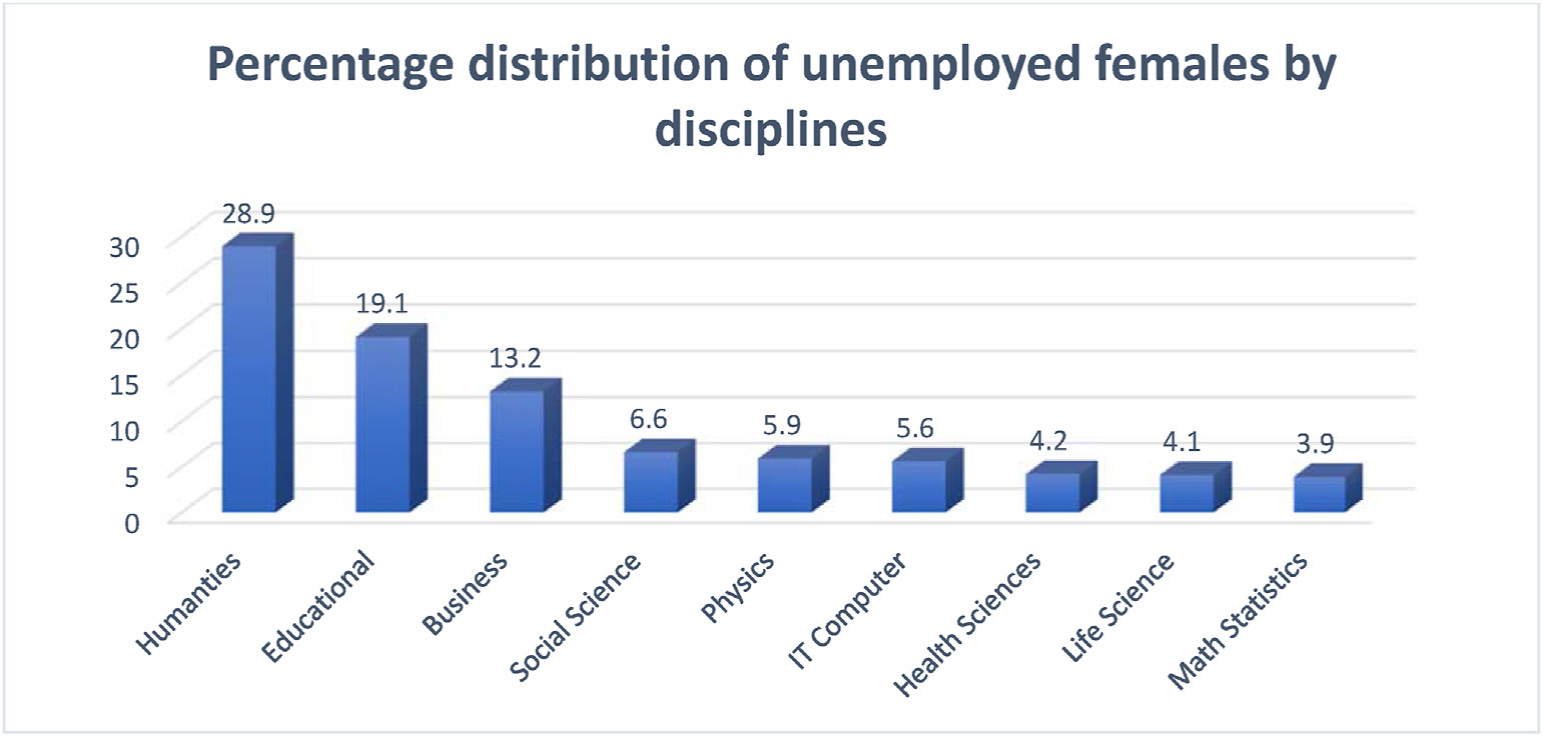
Percentage distribution of unemployed females by disciplines.

Source: Labor Force Participation reports of General Authority for Statistics (2010–2018)

The data presented in Figures 1, 2, 3, and 4 gave rise to the question about some specialties being more relevant to increasing female participation in the labor market. Figure 5 shows the percentage and distribution of unemployment among Saudi females with bachelor's degrees by disciplines.

Figure 5 shows that the unemployment rate among the female graduates of humanities and education is the highest, being 28.9% and 19.1% respectively, followed by business and social sciences. The unemployment rate is the lowest (between 6.6% and 3.9%) among the female graduates of scientific degrees such as physics, IT computer, health sciences, life sciences, mathematics, and statistics. This result suggests an increase in the employability of female graduates from natural science colleges; the specialties that contribute toward this surge are categorized as knowledge jobs, and hence this rate should be captured by the country's ranking under the KE Index. The following table explains what types of jobs absorb those graduates. Table 1 shows a positive and significant correlation between female graduates and the labor market.

**Table 1:** Correlation between female graduates and female job groups.

	Total Graduates	Science graduates	Health graduates	Technical graduates	Engineering graduates
Total Labor	0.902∗∗				
Specialists		0.299	0.451		
Technicians		-0.686∗	-0.695∗	-0.719∗	0.464
Engineers					0.618∗
Sales	0.954∗∗	0.555	0.920∗∗	0.800∗∗	0.556

Note: Table 1 also depicts a strong positive correlation between the graduates of health and technical specialties and the sales jobs.

∗significant at 5%.

∗∗significant at 1%.

Source: Own calculations: Labor Force Participation reports of General Authority for Statistics (2010–2018).

It is noteworthy to mention here that, according to many studies, sales-related jobs contribute mainly toward the knowledge economy, and salespersons are considered knowledge workers (Brinkley, 2008). However, Saudi women's participation in sales-related jobs is influenced by the labor market supply and demand. Sales jobs in the Saudi labor market cannot be characterized as knowledge jobs as that in the developed countries, where sales jobs are sophisticated, and the labor market is demanding and heavily reliant on technology, which is advanced in the service sectors (Reinhardt et al., 2011).

**Figure 6:**
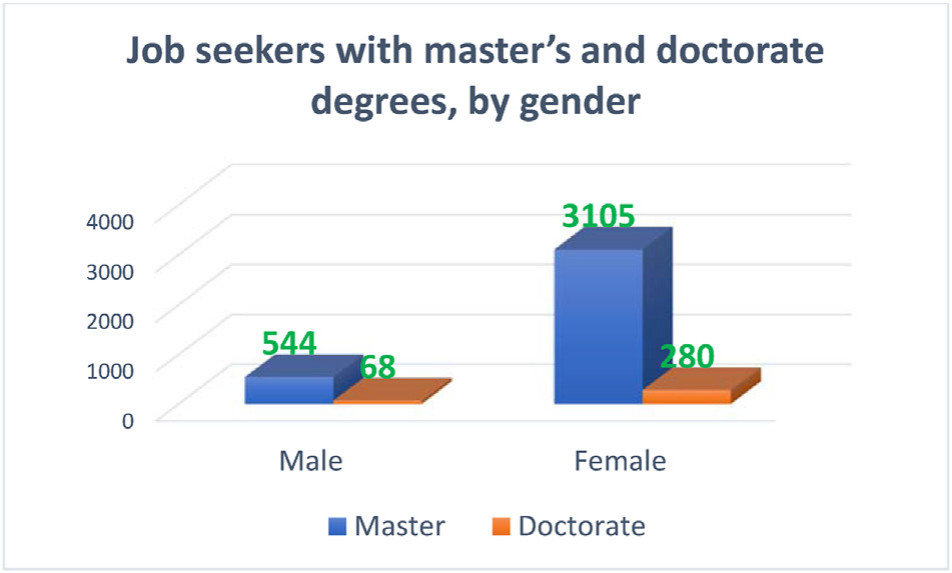
Job seekers with master's and doctorate degrees, by gender.

Source: Labor Force Participation reports of General Authority for Statistics

The discrepancy between the attained education at the level of a bachelor's degree and the labor market participation is amplified at the postgraduate level. This discrepancy can be observed in Figure 6, which shows the gender gap among Saudi Arabia's job seekers with master's and doctorate degrees.

Figure 6 indicates the number of unemployed females with master's degrees, which is almost four times higher than that of their male counterparts. Concerning the holders of the Doctorate degree, the figure shows that the unemployment rate among females is five times higher than that among males. This gender disparity highlights the importance of examining factors such as the pattern of growth and education attainment in the context of female labor force participation. Other factors such as household's economic conditions, social norms and the nature of economic growth, as suggested by Klasen (2019), should be investigated.

This section has exposed a clear gender gap and discrepancy between women's education and employment. As has been proved in previous studies in different contexts, KE can provide the best opportunity to the Saudi government to increase women's labor market participation and, consequently, to improve their GDP and accelerate the process of transition to a knowledge-based economy (Abd Elkhalek, 2017). Nevertheless, there is a lack of public gender-related information in relation to specific KE indicators, such as innovation and ICT, in the country. This may impact the country's transformation process, given the significance of role of big data in charting economic plans—evidence-based policymaking. It also highlights the importance of analyzing the nature of the available jobs in the labor market and its relationship with knowledge.

### 4.2 Human development index

**Figure 7:**
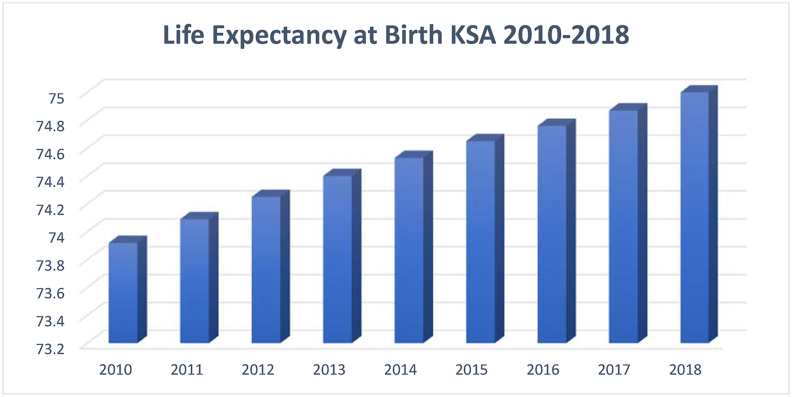
Life expectancy at birth KSA 2010–2018.

Source: The global economy (2018)

**Figure 8:**
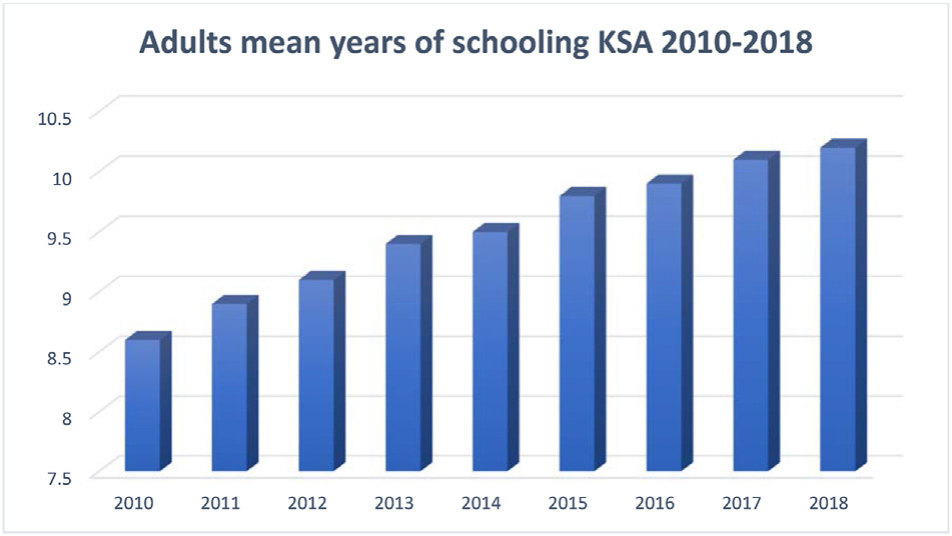
Adults' mean years of schooling KSA 2010–2018.

Source: The global economy (2018)

**Figure 9:**
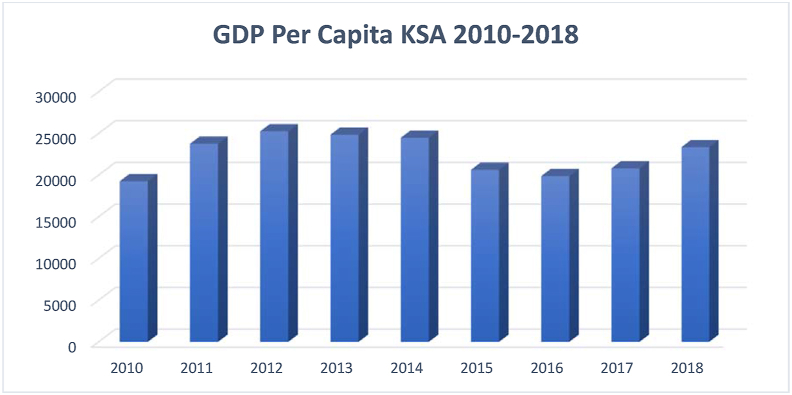
Gdp per capita KSA 2010–2018.

Source: The global economy (2018)

Human Development Index (HDI) is one of the most important indicators of KE, and it is closely related to education. Human development can be defined as "the knowledge, skills, competencies and other attributes embodied in individuals or groups of individuals acquired during their life and used to produce goods, services or ideas in market circumstances" (Organisation for Economic Co-operation and Development, 2007). HDI is the composite index measuring the average achievement based on three key components—health (measured by life expectancy at birth), education (measured by mean years of schooling for adults of 25 years and above), and decent standards of living (measured by the per capita income). The following Figures 7, 8, and 9 show Saudi Arabia's performance in the three dimensions of HDI for the sampled period.

Figures 7, 8, and 9 reflects constant rise in adults’ education and life expectancy in Saudi Arabi. GDP per capita, on the other hand, has witnessed a decline between the years 2015 and 2018. It is critical for countries aiming at a knowledge-based economy such as Saudi Arabia to invest in HD, particularly in higher education, which leads to innovation and accelerate progress toward a knowledge-based economy. In fact, researchers have associated HD with innovation and ICT adoption (Michie and Sheehan, 2003; Lopez-Garcia and Montero, 2012; Skorupinska and Torrent-Sellens, 2014).

Saudi Arabia's classification is considered satisfactory when it comes to HDI; however, this ranking may not capture Saudi government's goals in this area owing to different reasons. For instance, as one of the most influential members of the G-20 group and given its strong macroeconomic performance, Saudi Arabia is expected to exhibit a better and higher ranking. The influence of country’s performance, however, will be witnessed more profoundly in the section that discusses education and innovation.

**Figure 10:**
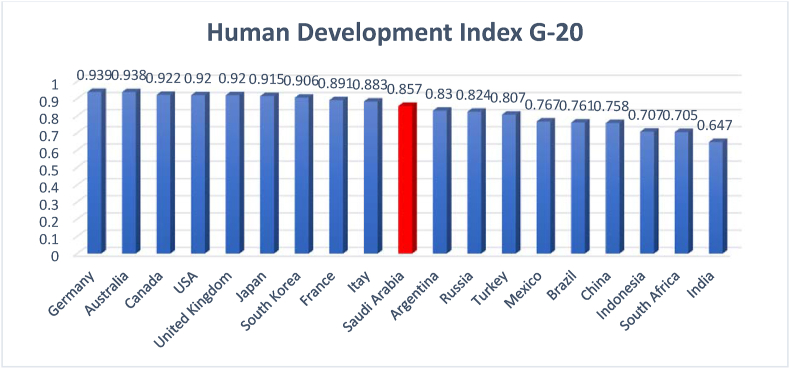
Human development index of the G-20.

Source: The global economy (2018)

Figure 10 shows the position of Saudi Arabia in the international HDI.

**Figure 11:**
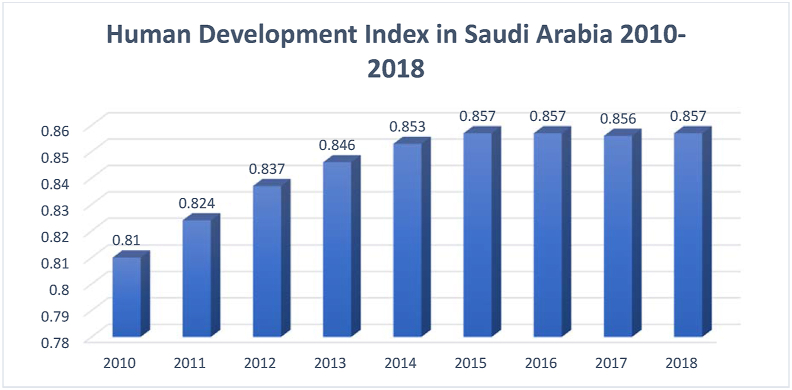
Human development index in Saudi Arabia (2010–2018).

Source: The global economy (2018)

Figure 10 illustrates the ranking of G-20 countries, according to the HDI performance in 2018. Saudi Arabia ranks in the 10^th^ position, next to Italy and before Argentina. This ranking indicates the good performance of Saudi Arabia in the HDI components. Figure 11 presents details regarding Saudi Arabia's HDI ranking over the sampled period.

The HDI indicator stands out among the other indicators discussed in this study. The HDI trend between 2010 and 2018 shows an increasing pattern, justifying the relatively high ranking of the country under this KE indicator. It is important to mention here that government spending and oil exports rather than KE are the main drivers of human development index (HDI) in Saudi Arabia (Haque and Khan, 2019).

**Figure 12:**
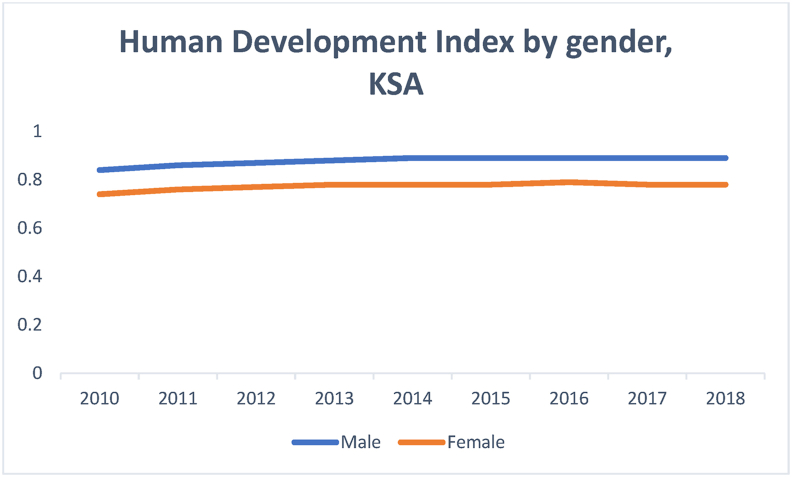
Human development index.

Source: The global economy (2018)

Figure 12 shows that Saudi Arabia has achieved its closest gender equality under HDI, following education; this reflects the importance of equality under KE and the better chances of the labor market inclusion of women.

The HDI trend based on gender shows a steady performance; however, there is a gender gap in favor of male, which is not statistically significant (0.89–0.78).

While Saudi has a high ranking under HDI, among the G-20 countries, this ranking is not reflected for the innovation index. HD is correlated with education and, consequently, innovation and ICT (Michie and Sheehan, 2003; Lopez-Garcia and Montero, 2012; Skorupinska and Torrent-Sellens, 2014). The relatively low ranking in innovation raises question over the efficiency of the output of the Saudi education system. The next section presents information on the most important indicators of KE in Saudi Arabia, without reference to gender. However, our data suggest a possible increase in women inclusion and the scope for improving the country's ranking in KE indexes.

### 4.3 Innovation and ICT

**Figure 13:**
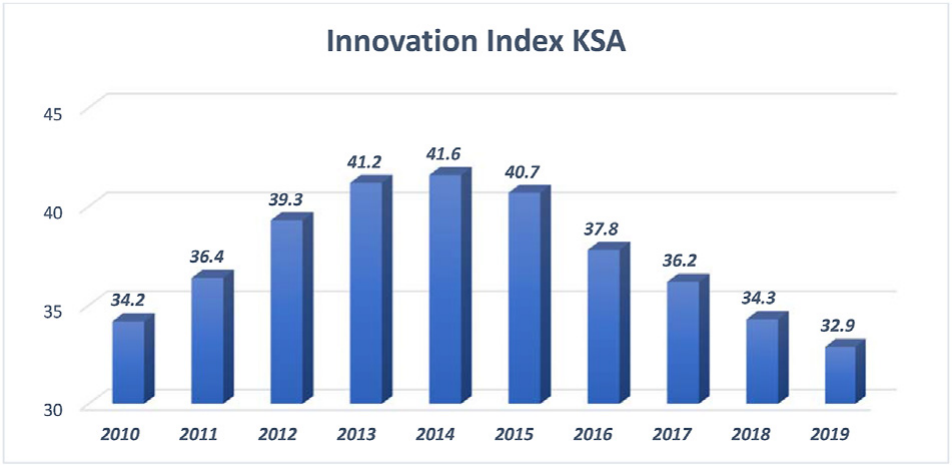
Saudi Arabia's innovation index between 2010 and 2019.

Source: The global economy (2010–2018)

This section presents details regarding Saudi Arabia's status in innovation, research and development and ICT. It also compares this status to the rest of the G20 countries to shed light on the areas where the country needs further improvement. Although Saudi Arabia has accomplished a remarkable advancement in its HDI ranking, its Innovation Index remains an area of concern. Figure 13 shows the trend of the country's Innovation Index for the period 2010–2018.

#### 4.3.1 Innovation index

**Figure 14:**
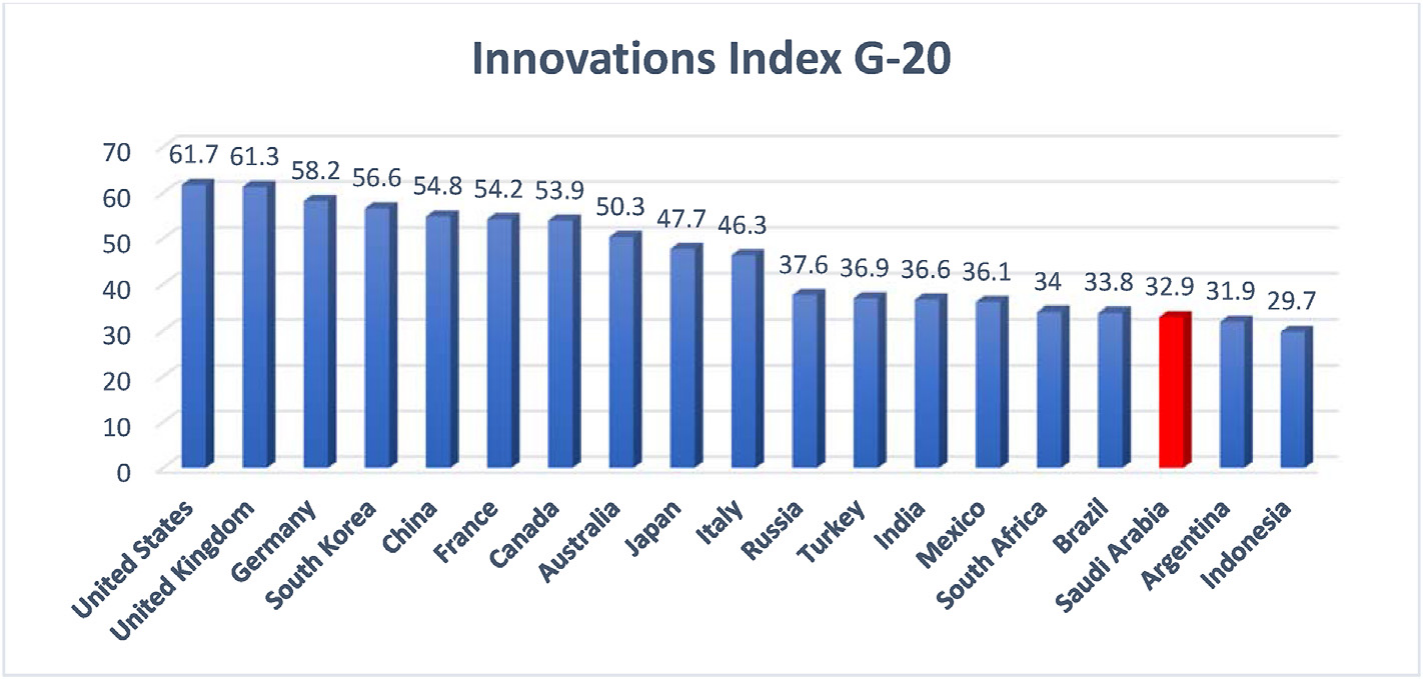
Innovation index of the G20 countries.

Source: The global economy (2018)

The 2010–2014 data regarding innovation in Saudi Arabia show a gradual increase in the country's ranking on the international innovation index. The increase, however, was followed by a constant decline in the curve between 2015 and 2019, as depicted in Figure 13. The decline in the international innovation index has impacted Saudi Arabia's ranking among the G20 countries (see Figure 14).

**Figure 15:**
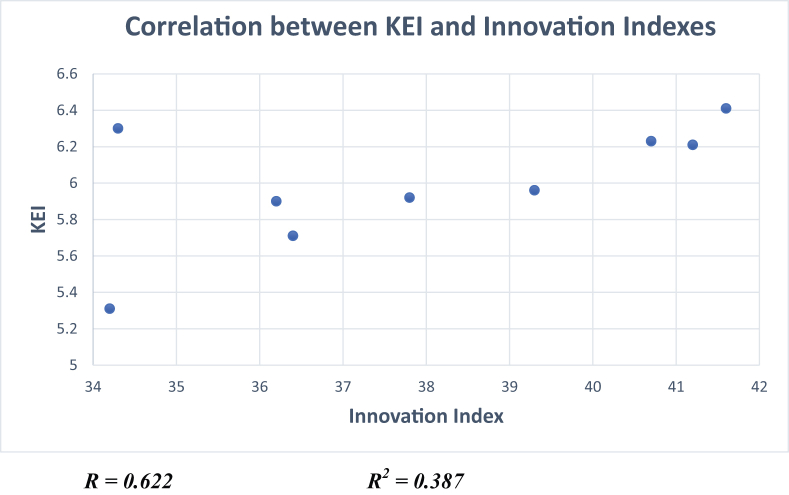
Correlation between innovation index and KE indexes.

Source: Own Calculations from Global Economy Statistics

It is agreed upon by scientists and economists that innovation is vital for countries striving to evolve into a knowledge-based economy. Saudi Arabia's low ranking in innovation has negatively impacted the country's international ranking owing to the strong correlation between innovation and KE indexes, as can be seen in Figure 15.

The analysis of the relationship between the KE and innovation indexes revealed a positive and significant correlation between the two indicators, as reflected in Figure 15. This correlation stresses the importance of investigating innovation to improve the country's KE index status. An in-depth examination of the components of innovation and related elements, such as patents and development and research expenditure, shows Saudi's low ranking in research and development expenditure, which partially explains its low ranking in the innovation indexes. It is important to mention that the gender-related data were missing from the public sphere, an issue that hinders an understanding of women's participation in the KE.

**Figure 16:**
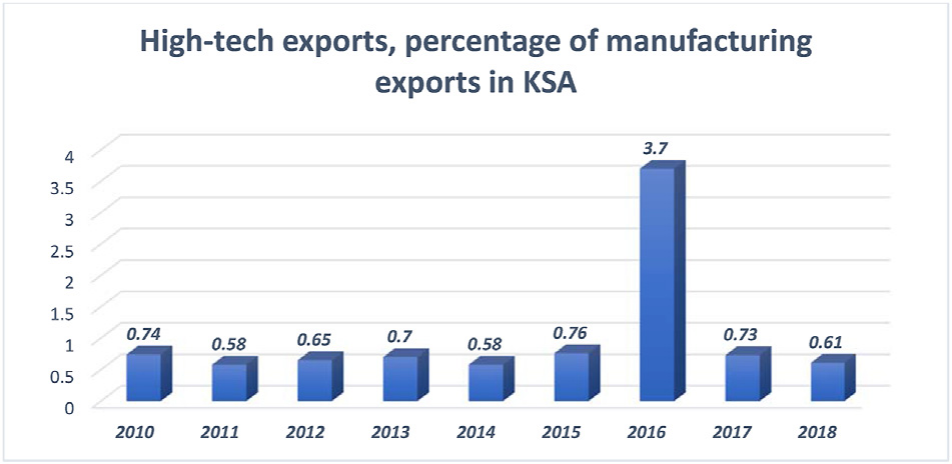
High-technology exports as a percentage of manufacturing exports.

Source: The global economy (2010–2018)

The percentage of high-technology exports is one of the indicators of a country's innovation status. This indicator significantly impacts the country's positioning among the knowledge-producing countries and its economic performance. Figure 16 shows the trend of high-tech exports in Saudi Arabia.

**Figure 17:**
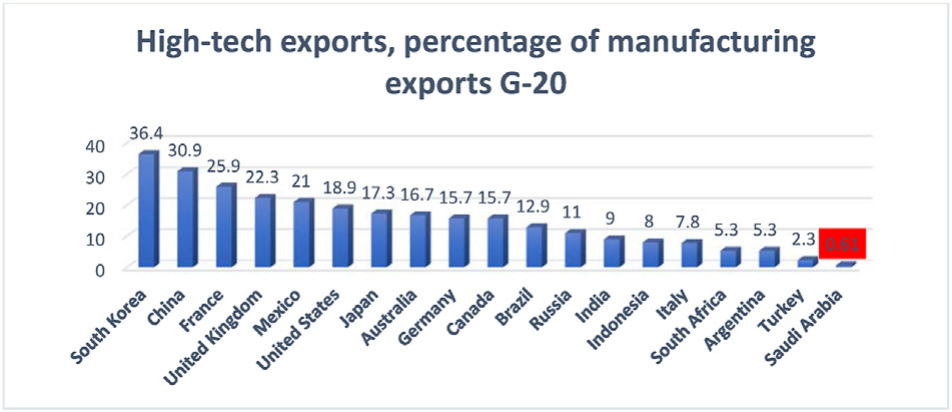
G20 countries' high-technology exports as percentage of their manufacturing exports.

Source: The global economy (2018)

Figure 16 reflects a low percentage of high-technology exports during 2010–2018. However, 2016 witnessed a five-fold increase in the percentage of high-technology exports. This percentage was not maintained in the subsequent years—2017 and 2018. In fact, it drastically dropped to a lower percentage—0.73 and 0.61 in 2017 and 2018, respectively. This low percentage impacted the country's ranking among the G20 countries (see Figure 17).

Patent application is another element of innovation that significantly contributes toward the innovation index ranking. The following chart shows the trend of patent applications by Saudi residents.

**Figure 18:**
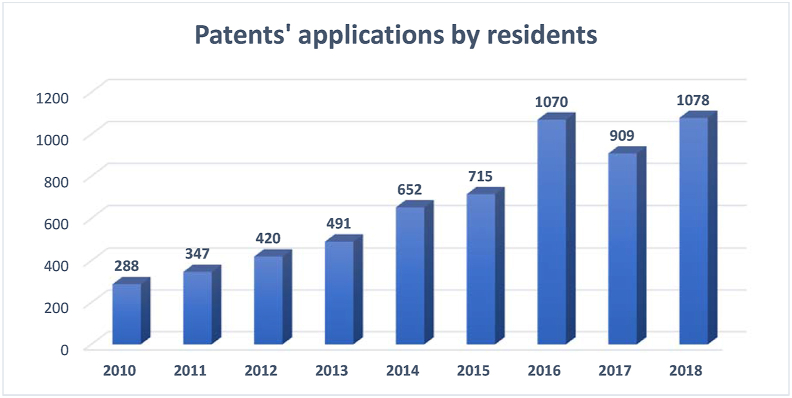
Patents applications by Saudi Arabia residents.

Source: The global economy (2018)

**Figure 19:**
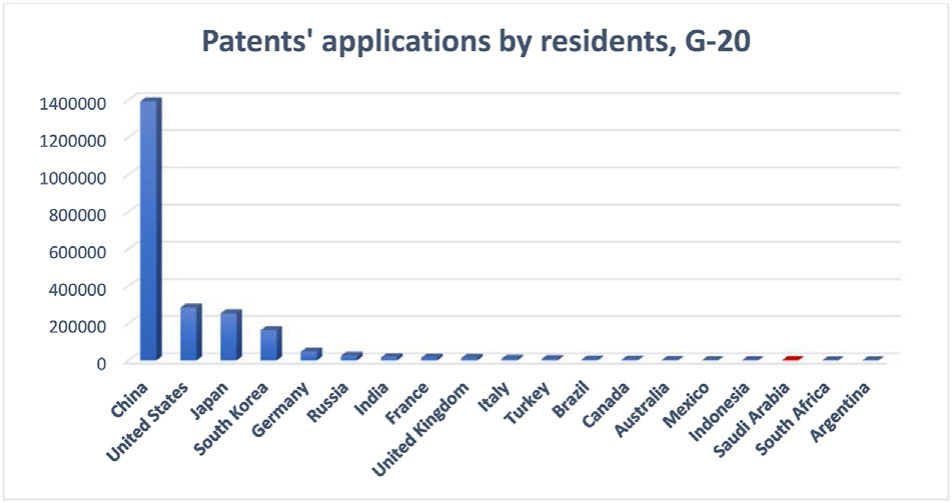
Patent applications by G20 countries' residents.

Source: The global economy (2018)

Figure 18 shows a constant increase in patent applications during 2010–2018. This increase was not parallel with the high-technology exports of the country, as depicted in Figure 17. This disparity between the number of patent applications and the high-technology exports as a percentage of manufacturing exports indicates a possible gap between knowledge production and knowledge utilization (Sandu and Bogdan, 2014). The increase is not comparable to the achievement of the other G20 group members (see Figure 19).

Patents are important indicators of the direction of innovation in a country. However, when patents and inventions are converted into innovation, they create "patent-sensitive exports" that add significant value to the country's economy (Ivus, 2010). The primary function of patents "is to provide incentives for invention and possibly to promote the diffusion of knowledge" (Hall, 2020, 1). However, in reference to the Saudi context, the inability of the patents to be translated into innovation raises questions on the nature of the patents and its relationship with high-technology versus low-technology manufacturing. It also questions the relationship between education and the nation's high-technology industry.

**Figure 20:**
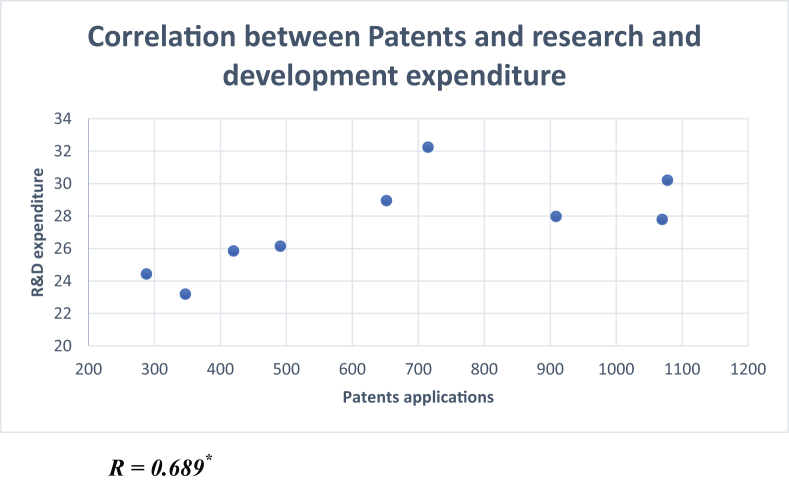
Correlation between patents and the research and development expenditure.

Source: Own Calculations from Global Economy Statistics

Research development and expenditure are also strongly related to patents and, consequently, innovation. However, the relationship is quite complex as it involves other elements such as higher educational institutions, technology use, and the industry itself (Ivus, 2010; Altbach, 2013). Figure 20 depicts the correlation between patents and the research development expenditure in Saudi Arabia.

Figure 20 shows a significant and positive correlation between research and development expenditure and patent applications. The value of correlation coefficient was calculated at 0.689 for 2010–2018, as can be seen in the above scatterplot. This positive correlation will significantly impact the country's high-technology products and intellectual capacities (Sandu and Bogdan, 2014). This positive correlation is not captured by the country's general ranking in the G20 group.

#### 4.3.2 Correlation of ICT indicators with patents applications in Saudi Arabia

ICT occupies a central place in the KE—it is considered as the driving force of the KE. However, there recently have been increasing calls for patent-based analyses to better understand the ICT as indicator and to foster development of the KE (Liu et al., 2020). Concerning its status in Saudi Arabia, the country's heavy investments in ICT have helped it secure a relatively high ranking for several ICT components, such as network readiness and individual usage. However, Saudi Arabia is still struggling with transferring its ICT and patent capacity to advanced technological innovation, as planned in its Vision 2030.

To investigate the reasons behind the current status of Saudi Arabia in relation to its ICT determinants and its relationship with patents' application and innovation, the researchers built the regression model described below.

##### 4.3.2.1 Correlation matrix of innovations, patents, and ICT determinants

Selected variables:

• Innovation Index
• Patents applications by residents
• Research and development expenditure, percentage of GDP
• Internet use, percentage of the population
• High-tech exports, percentage of manufacturing exports
• Mobile phone subscribers, in millions
• Capital investment, percentage of GDP.

The correlation matrix above (see Table 2) summarizes the results of the Pearson correlation coefficients of the selected innovations, patents, and ICT determinants. Patent applications were positive and significantly correlated with the research and development expenditure. Patent applications were also found to be significantly correlated with Internet use as a percentage of the population. A negative significant correlation was identified between patent applications and mobile phone subscribers. The use of the Internet as a percentage of the population had a positive and significant correlation with research and development expenditure. The indicator of capital investment had a positive and significant correlation with high-technology exports as a percentage of manufacturing exports. The result of the regression model indicates a problem in the country's national innovation systems and in the utilization of the strong ICT infrastructure for utilizing and disseminating knowledge. It also highlights the missing interaction between the producers and the users (Godin, 2003).

**Table 2:** Correlation matrix of innovations, patents, and ICT determinants.

	Innovation Index	Patent applications	R&D expenditure	Internet use	High-tech exports	Mobile subscribers	Capital investment
Innovation Index	1.0						
Patent applications	-0.129	1.0					
R&D expenditure	0.293	0.689∗	1.0				
Internet Use	-0.092	0.945∗∗	0.743∗∗	1.0			
High-tech exports	0.020	0.556	0.113	0.280	1.0		
Mobile subscribers	0.563	-0.766∗	-0.354	-0.808∗	-0.148	1.0	
Capital investment	-0.033	-0.042	0.142	-0.067	0.697∗	-0.209	1.0

∗ significant at 5%.

∗∗ significant at 1%.

Innovation, patents, and research expenditure are usually associated with higher education outputs. The education provided by universities is part of the "innovation system," which is discussed by Chen and Dahlman (2004, 9). Therefore, it is important to understand the relationship between Saudi's higher education system and those elements. The next section investigates the relationship between innovation, patents, and research in Saudi Arabia and relates it to the education output and the KE. This approach represents a step toward investigating the possibility of gendering the KE, using the available information regarding the gender dimension under the education indicator (discussed in Section 4.1).

#### 4.3.3 Innovations and patents correlation with higher education output in Saudi Arabia

Owing to the difficulty in obtaining information about gender in the context of innovation, patents and ICT, the research team examined the relationship between education and these indicators. Innovation and patents are usually closely correlated with education's outcome. They also play a crucial role in the country's ranking in the KE. Therefore, when moving to a knowledge-based economy, the government emphasizes specialties such as natural sciences, ICT, and mathematics (i.e., STEM). However, such specialties are not strategically planned in the Saudi context as graduates of those specialties, according to our data, have been absorbed by sales-related jobs (see Table 1). This finding highlights the importance of conducting an in-depth examination of the education system and its role in the national innovation system. An investment in STEM graduates will contribute toward reducing the unemployment rate and increasing the GDP in the country.

Table 3 summarizes the relationship between innovation, patents, and local postgraduate education in Saudi Arabia, for the period 2018–2018.

**Table 3:** Correlation between the innovation index and patents and local postgraduates.

	Innovation		Patents	
Graduates	R	P-value	r	P-value
Doctorates total	-0.379	0.314	0.065	0.868
Doctorates male	-0.316	0.408	0.566	0.112
Doctorates female	-0.129	0.741	-0.673	0.047
Master’s total	0.148	0.705	0.787	0.012
Master’s male	0.086	0.827	0.820	0.007
Master’s female	-0.057	0.885	0.819	0.007

Source: Own Calculations from Global Economy and Ministry of Education Statistics

The results show that patent applications by residents were significantly and positively correlated with local postgraduates for all genders (P < 0.05). The same finding was not arrived at regarding doctorates in relation to patents. There is no significant correlation between the holders of master's and doctorate degrees and innovation. This interesting result highlights the importance of examining the nature of the education of those postgraduates.

To better understand the relationship between the nature of education and innovation and patents, the researchers considered the local graduates in science and information technology (IT) (Tables 4 and 5).

**Table 4:** Correlation between the innovation index and patents and local science postgraduates.

	Innovation		Patents	
Graduates	r	P-value	r	P-value
Doctorates total	-0.421	0.259	0.047	0.904
Doctorates male	-0.543	0.131	0.373	0.323
Doctorates female	-0.255	0.508	-0.176	0.650
Master’s total	-0.438	0.238	0.296	0.439
Master’s male	-0.202	0.603	-0.213	0.582
Master’s female	-0.468	0.204	0.436	0.240

Table 4 shows no significant correlation between the local science postgraduates and innovation and patents during the sampled period. This result is another indicator of a problematic national innovation system. It also highlights the missing link between the universities, government, and industry, as explained by the triple helix approach (Shin et al., 2012).

Table 5 shows the positive significant correlation between the local postgraduates of IT and the patents, but no significant correlation between the postgraduates and innovation was found. The number of doctorates in IT is significantly low owing to the lack of such programs in local universities. The first PhD program started in 2015.

**Table 5:** Correlation between innovation index and patents and local IT postgraduates.

	Innovation		Patents	
Graduates	r	P-value	r	P-value
Master’s total	-0.375	0.320	0.641	0.045
Master’s male	-0.406	0.279	0.633	0.043
Master’s female	-0.342	0.367	0.611	0.048

Table 6 shows the relationship between the Saudi postgraduates from international universities and innovation and patents. The table shows, like with the local graduates, a significant and positive correlation between the offshore postgraduates and doctors and patents. There was no significant correlation between the offshore postgraduates and doctors and innovation. Nevertheless, the value is positive compared to the local graduates, where the (r) value was negative; it implies that a positive correlation does not rise to the level of being statistically significant.

**Table 6:** Correlation between the innovation index and patents and offshore postgraduates.

	Innovation		Patents	
Graduates	r	P value	r	P value
Doctorates total	0.220	0.569	0.922	0.000
Doctorates male	0.308	0.419	0.883	0.002
Doctorates female	0.061	0.877	0.967	0.000
Master’s total	0.540	0.134	0.722	0.028
Master’s male	0.624	0.073	0.644	0.061
Master’s female	0.392	0.297	0.818	0.007

## 5 Conclusion

The increasing international emphasis on knowledge as a driving force for economy, wealth, and job creation has directed Saudi Arabia's pursuits toward developing a high-skilled and high-value economy. The Saudi government's realization of the growing value of knowledge as an economic asset has also led to a strong emphasis on transitioning from an oil-based to a knowledge-based economy (Bafarasat and Oliveira, 2021; Onsman, 2010). This realization was also associated with the determination to empower women and place them at the center of the country's educational and economic transformation plans in order to increase their participation in the labor market to 30% by 2030. Despite this importance placed on women's economic participation, Saudi women's labor market participation is less than satisfactory. According to the available literature, the KE presents the best answer to both women's inclusion in the formal economy and a country's economic acceleration.

This study is a step toward having a better understanding of Saudi women's status in relation to the five most critical KE pillars—education, employment, HDI, innovation, and ICT, the first research objective. The findings confirm that the Saudi government is on the right path with its initiative to empower women through education. Education has witnessed a high percentage of women enrollment, especially at post-secondary level, which is considered as the bridge to the labor market. Nevertheless, a discrepancy was noted between women's education and employment. With a female unemployment level of more than 30%, the Saudi government is faced with great challenges. Surprisingly, more than 40% of the unemployed Saudi female graduates are bachelor's degree holders, who can be enlisted under the umbrella of the KE (i.e., STEM). Hence, to improve its current low ranking on KE indexes, improve the GDP and transition to a knowledge-based economy, the Saudi government should revisit unemployment in general and among women in particular and introduce appropriate actions to change the status quo. These efforts should be directed toward rehabilitating unemployed women, reskilling/upskilling them, especially with STEM-related degrees, and including them in KE-related jobs.

In this context, it must be noted that job creation under the KE is not a simple, straightforward process. It is a complicated interaction between different actors including universities, government, and industry. The researchers have noted an inconsistency in the Saudi ranking under the pillars of the KE; while Saudi Arabia has a good ranking in education, HDI, and most of the ICT enablers, its ranking in innovation and patents is poorer than most of the G-20 countries (Index of Knowledge Economy, 2018). This finding reflects the research's second objective.

Importantly, for a country struggling to increase women's labor market participation, not attending to the gender dimension under ICT and innovation is quite alarming. The lack of correlation between the education output and the vital elements of the KE, such as patents and innovation, indicates a missing link in and deviation from the long-established relationship between education and innovation and between innovation and GDP. This study highlighted the lack of higher education's contribution toward innovation, the answer to the third research objective.

## 6 Implications

The study concludes with several recommendations that can be summarized as follow:

1. The Saudi government should emphasize the role of higher education and increase the competitiveness within its establishments. This can be done by introducing a system of incentives that accelerates the process of knowledge production and dissemination.
2. The Saudi government should also utilize its robust ICT infrastructure and ensure ICT adoption.
3. It should view innovation as an end-to-end process that starts at educational establishments and ends in the local and international markets.
4. Given this, the government should create a sophisticated knowledge market that can absorb the higher education outcome and utilize it.
5. Saudi Arabia must invest in HD, particularly in higher education, which leads to innovation and accelerates the country's transition toward a knowledge-based economy.
6. The lack of public gender-related information under specific KE indicators (e.g., innovation and ICT) will impact the country's transformation process, given the significance of big data in charting future economic plans—evidence-based policymaking.
7. There is an urgent need to carry out a nationwide survey to analyse the nature of the available jobs in the labour market and their relation to knowledge. Understanding the nature of the currently available and the needed jobs is a critical step towards matching them with the output of higher education. It also presents an essential step in providing relevant training to reskill or upskill the HE graduates.

## Declarations

### Author contribution statement

Sabria Jawhar; Sajjadllah Alhawsawi; Asaad Jawhar; Mohmmad E. Ahmed: Conceived and designed the experiments; Performed the experiments; Analyzed and interpreted the data; Contributed reagents, materials, analysis tools or data; Wrote the paper.

Kholoud Almehdar: Performed the experiments; Contributed reagents, materials, analysis tools or data.

### Funding statement

Dr. Sabria Salama Jawhar was supported by Ministry of Education [RJ20/009/J].

### Data availability statement

Data included in article/supp. material/referenced in article.

### Declaration of interest’s statement

The authors declare no conflict of interest.

### Additional information

No additional information is available for this paper.
